# “O. U. C.” H.

**Published:** 1864-09

**Authors:** 


### Correspondence.

### "O. U. C." H.

"For grief was heavy at his heart,
And tears began to flow."

"He went to his chamber—his chamber and wept."

BUT they were bright cheerful fellows, My Dear DENTAL REGISTER, in comparison with an O(ld) U(gly) C(roaker) that I knew in my boyhood—merry chaps, indeed, when compared with your O(ld) U(ntiring) C(ontributor), who used to refresh your pages with his monthly "gossip." "*What is the matter with him?*" "O. U. C." my dearest "REGISTER," O(ur) U(ncle) C(oncealed), as a certain firm might have called him till recently, "has come to grief." Yes, even a step further. He is surprised—yea, verily, "more than surprised." What! O(ur) U(ncle) C(onfounded)! "Ponder it—think of it," O my REGISTER, and then you will

"Lament in rhyme, lament in prose,
Wi' saut tears tricklin' down your nose;"

and you will join this O(ld) U(nceasing) C(omplainer) in chanting his Jeremiad. "Is it nothing to you, all ye that pass by? behold and see if there be any sorrow like unto my sorrow!"

And now REGISTER, though I love you as if you were my own son, I fear you are not guiltless of this grief. I must talk plainly to you. Do you always treat your comrades with courtesy. And especially, did you "notice" the birth of your young relative, in the Quaker City, a year ago? I saw, on your Editors' table, a very flattering notice of the interesting event; but did you get it into print? Here, in the mountains, I am not able to answer for you; but if you didn't, just apologize.

And REGISTER, haven't you been naughty in neglecting to give due credit? Be careful. But I will try to make allowance for errors of the head, while I know your heart is right. I will try to excuse oversights, in view of the weight of your war subscription. I will remember that you gave the life blood of your proprietor and publisher, as a sacrifice on your country's altar—that you spared one of your editors, a business assistant, most of your special contributors, and many, very many, of your subscribers, to the service of your country. Those left to labor for you are, consequently, overworked to such extent that minor mistakes may occur.

But why should these little shortcomings excite O(ur) U(ncle's) C(holer)? you ask. "O. U. C.," the journal you probably failed to notice, is the organ of O(ur) U(ncle's) C(ollege) that college he used to advertise in your pages, *under the disguise of special correspondence.* 'Tis *that* that makes O. U. C(ry).

And now, REGISTER, you and I may never meet again, and hence I may give you a little advice. When you undertake to criticise, do not allow yourself to be either "surprised" or "grieved;" for then you might be so reckless as to murder the whole science of rhetoric in a few sentences. As proof that such a crime may be committed, see DENTAL TIMES, VOL. 2, p. 20, 21. W.

